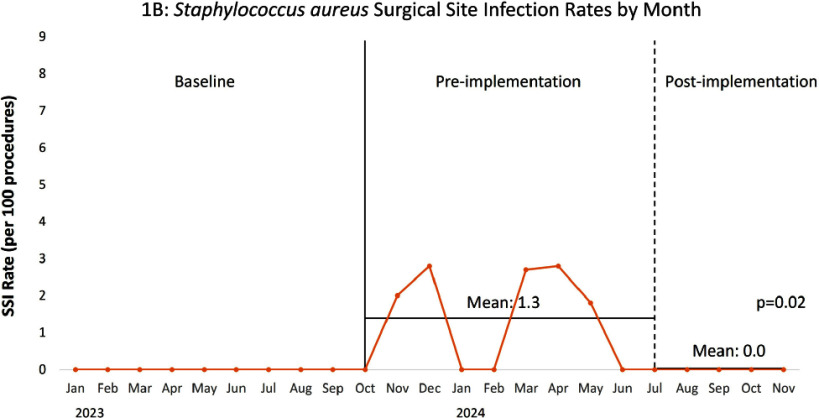# Preoperative Nasal Povidone Iodine to Prevent Staphylococcus aureus Surgical Site Infections in Pediatric Patients

**DOI:** 10.1017/ash.2025.410

**Published:** 2025-09-24

**Authors:** Ashley Lloyd, Emily Hunter, Adriana Condren, Derek Harford, Angela Niesen, Elizabeth Daniels, Patrick Reich

**Affiliations:** 1 St. Louis Children’s Hospital; 2 Saint Louis Children’s Hospital; 3 BJC; 4 St. Louis Children’s Hospital; 5 Washington University in St. Louis; 6 Washington University School of Medicine

## Abstract

**Background:** From October 2023-June 2024 increased surgical site infection (SSI) rates were identified in our large pediatric hospital, 38% were caused by Staphylococcus aureus. Nasal S. aureus colonization is associated with increased SSI risk and preoperative nasal decolonization decreases S. aureus SSI risk. Historically, our institution recommended a five-day course of nasal mupirocin decolonization prior to selected high-risk procedure types, though this process it not possible for urgent cases and outpatient compliance is low. Nasal povidone iodine (PI) is a topical antiseptic used commonly in adults as an alternative to nasal mupirocin for S. aureus decolonization and SSI prevention. This practice is less commonly described in pediatric patients. **Methods:** In addition to standard SSI prevention measures, universal nasal PI application was implemented preoperatively (as a single topical application following induction of anesthesia) in July 2024 for patients ≥34 weeks corrected gestational age (CGA) undergoing the following high-risk surgical procedures: ventricular shunts, spinal fusions, and all cardiothoracic (CT) procedures. Compliance with nasal PI application was monitored based on documentation in the electronic medical record. Mean monthly total SSI rates (per 100 procedures) and mean monthly S. aureus SSI rates for these procedure types were followed pre- and post-implementation of universal nasal PI and compared via unpaired t-test. **Results:** Documented compliance with nasal PI application was 51% overall, ranging from 22% for ventricular shunts to 75% for CT procedures. Implementation of universal nasal PI preoperatively was associated with a non-statistically significant decrease in composite mean SSI rates (Figure 1A): 3.5 per 100 procedures pre-implementation, 2.3 post-implementation (p=0.52). A statistically significant decrease in composite mean S. aureus SSI rates was observed (Figure 1B): 1.3 per 100 procedures pre-implementation, 0.0 post-implementation (p=0.02). **Conclusion:** Despite modest documented compliance, implementation of a universal preoperative nasal PI program, in conjunction with standard SSI prevention measures, was associated with decreased S. aureus SSI rates in pediatric patients undergoing high-risk surgical procedures.

Figure 1. Total (1A) and Staphylococcus aureus (1B) surgical site infection (SSI) rate per 100 ventricular shunt, spinal fusion, and cardiothoracic procedures (combined) by month from January 2023 through November 2024. The solid vertical line indicates the beginning of the period with increased S. aureus SSI rates (pre-implementation period). The dashed vertical line indicates the start of the implementation period. Mean SSI rates for the pre- and post-implementation periods are indicated by the horizontal lines and compared via t-test.

**Figure:**
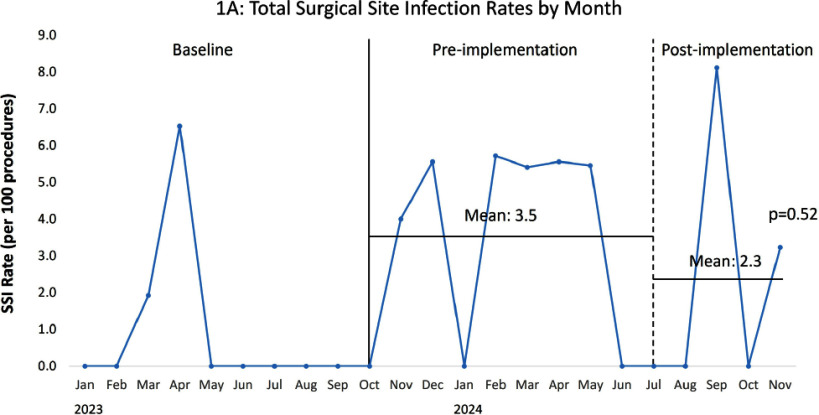

**Figure:**